# The First Identification and Antibiogram of *Clostridium perfringens* Type C Isolated from Soil and The Feces of Dead Foals in South Korea

**DOI:** 10.3390/ani9080579

**Published:** 2019-08-20

**Authors:** Chul Song Park, Ji Yong Hwang, Gil Jae Cho

**Affiliations:** Laboratory of Equine Medicine, College of Veterinary Medicine and Institute of Equine Medicine, Kyungpook National University, 80, Daehak-ro, Buk-gu, Daegu 41566, Korea

**Keywords:** *Clostridium perfringens*, *beta*-toxin, 16S rRNA, antibiotic, South Korea

## Abstract

**Simple Summary:**

*Clostridium (C.) perfringens* in horses causes acute enteritis and death, but research on *C. perfringens* in South Korea is virtually nonexistent. The purpose of this study was to discover the cause of death of numerous South Korean foals. *C. perfringens* was isolated from 25 (11.1%) of 225 sampled horses and from 16 (35.56%) of 45 farms investigated in this study. Of the 25 *C. perfringens* isolates, 15 (60%) were type A and 10 (40%) were type C. Type C was observed on all the farms where the foals’ deaths occurred. The antimicrobial susceptibility test was performed using MIC (Minimum Inhibitory Concentration) Evaluator strips test. These results are the first to identify one of the causes of acute foal death in South Korea and can be used as a criterion to determine the cause of acute foal death and to select effective antibiotics.

**Abstract:**

*Clostridium (C.) perfringens* was isolated from 25 (11.1%) of 225 sampled horses and from 16 (35.56%) of 45 farms. All of the samples were negative for *cpe,* *etx*, *itx, NetF* genes and *cpa* *gene* were detected in 100% (25 of 25) of the samples that were positive for *C. perfringens*. *cpb* and *cpb2* were detected in 40.0% (10 of 25) and 60.0% (15 of 25) of the samples that were positive for *C. perfringens*, respectively. Of the 25 *C. perfringens* isolates, 15 (60%) were type A and 10 (40%) were type C. Type C was observed on all the farms where the foals’ deaths occurred. None of the isolates were positive for type B, type D, or type E. The MIC Evaluator strips antimicrobial susceptibility test showed meropenem (96%), ampicillin (92%), amoxicillin/clavulanic acid (84%), and tetracycline (8%) sensitivity.

## 1. Introduction

*Clostridium (C.) perfringens* is an anaerobic, endospore-forming, gram-positive, rod-shaped bacterium that is ubiquitous in various environments, especially in soil and the gastrointestinal tract of humans and animals [1,2,3,4,5,6]. *C. perfringens* causes numerous gastrointestinal infections in most mammalian species. These infections are generically called enterotoxemia because toxins produced in the intestine may be absorbed into general circulation [7,8]. *C. perfringens* produces at least 17 different toxins and, according to the production of the major toxins *alpha, beta, epsilon,* and *iota*, it has been classified into five different [8,9] types. Although the diseases caused by each toxin are somewhat different, we hypothesized that the *beta*-toxin of *C. perfringens* type C can cause death in foals. *C. perfringens* type C produces the highly necrotizing and lethal *beta*-toxin, which is responsible for severe intestinal necrosis. The *beta*-toxin is encoded by the *cpb* gene, which is located on a plasmid [3]. The *beta*-toxin induces lethal infections ranging from necrohemorrhagic enterocolitis to enterotoxemia in pigs, cattle, sheep, and goats and in the neonatal animals of numerous domestic animal species [4,10].

Studies on *C. perfringens* are currently underway in the world [3,4,5,11,12,13]. In particular, research on *C. perfringens* type A and its bacterial effects on chickens is abundant, but there are not many studies on *C. perfringens* type C [1,8,9,14], and those in horses are even rarer [2,15].

*C. perfringens* type C is an important cause of enteritis and enterocolitis in foals and, occasionally, in adult horses [2]. Because *beta*-toxin is highly susceptible to the action of trypsin, neonatal animals are particularly susceptible to type C infections due to the low level of intestinal trypsin in the first days of life and the presence of trypsin inhibitors in colostrum [2]. The aim of this study was to identify *C. perfringens* and its toxin types under the hypothesis that *C. perfringens* was the cause of severe colitis in numerous foals in South Korea. We also performed antibiotic susceptibility tests for effective disease treatment.

## 2. Materials and Methods

### 2.1. Sampling

From 2009 to 2017, 225 horse fecal and soil samples were collected from 45 horse farms located on Jeju island and an inland region of South Korea. Seven samples were collected from the dead foals’ feces (foals who died of acute diarrhea within 3 to 5 days of birth) and contaminated soil around the dead body on each farm. If there were no dead foals on the farm or horse riding track without foals, then samples were collected from healthy horse feces and general soil for comparison (Table 1).

The symptoms of all the dead foals were the acute onset of diarrhea with multiple clinical signs of toxemia, such as fever, depression, dehydration, tachycardia, prolonged capillary refill time, lying down, congested mucous membranes, and multifocal hemorrhagic inflammations of the intestine identified by postmortem examination. These symptoms appeared within 5 days of the foal’s birth (data not presented here).

The collected samples were stored in sterile plastic tubes and frozen at −70 °C within 6 to 12 h for later analysis [16,17,18,19].

### 2.2. C. perfringens Detection

An attempt was made to isolate the causative bacteria from the samples of suspected farms to diagnose whether the cause of the foals’ deaths was *C. perfringens*. Samples were identified according to the previous study method [20]. First, 10 g of fecal or soil samples were collected in a new tube and diluted with 10 mL of 0.1 M phosphate-buffered saline (PBS, pH 7.4) (Gibco, Dublin, Ireland). The diluted tubes were shaken for 30 min using a shaking incubator (Vision Scientific, Seoul, South Korea). The supernatant (1.5 mL) was transferred to an Eppendorf tube and centrifuged at 1000 rpm for 5 min (Labogene, Seoul, South Korea). After centrifugation, 1 mL of the supernatant was taken out, transferred to another Eppendorf tube, and centrifuged at 8000 rpm for 10 min. After that, 1 mL of the supernatant was removed and 1 mL of PBS was added, transferred to a dry bath (Biometra, Dublin, Ireland), and heat treated at 80 °C for 10 min. The heat-treated sample (1 mL) was inoculated into cooked meat medium (CMM) (Oxoid, Hampshire, UK) and incubated in an anaerobic jar ( Rectangular jar, AnaeroPack ™, Thermoscientific, Massachusetts, US) at 37 °C for 2 days in an incubator (Panasonic, Osaka, Japan). The cultured CMM medium was shaken well, sub-cultured onto blood agar plates, and anaerobically incubated for 2 days at 37 °C. Two days later, the *C. perfringens* colonies were evaluated for the large double zone of hemolysis and microscopic morphology characteristics. These isolates were characterized using VITEK^®^ MS (Biomerieux, Marcy-l’etoile, France) according to the manufacturer’s instructions. They were stored in Microbank (Prolab, Texas, US) and further characterized as described in a later section.

### 2.3. Preparation of Genomic DNA

The DNA extraction of *C. perfringens* requires the extraction of its plasmid genes along with the chromosomal genes, because most of the toxin genes are derived from plasmids. Therefore, three DNA extraction methods were performed to obtain accurate results. The first was the boiling method in which one or two single colonies were added to 100 μL of distilled water or PBS, shaken thoroughly with sterilized platinum, heat-treated at 100 °C for 30 min in a dry bath, and centrifuged at 13,000 rpm for 5 min. PCR was performed using 50 μL of the supernatant [13,21]. Two other methods were performed using commercial DNA kits: Qiamp DNA mini kit (Qiagen, Hilden, Germany) and Qiaprep spin miniprep kit (Qiagen, Hilden, Germany), according to the manufacturers’ instructions.

### 2.4. 16S rRNA Gene Sequencing and Statistical Analysis

16S rRNA gene sequencing was performed for a more accurate identification of all the strains primarily identified as *C. perfringens* [22,23] with an ABI PRISM 3730XL Analyzer (Applied Biosystem, Foster City, CA, USA). The alignment was performed with BioEdit, and the nucleotide sequences of the strains were aligned using the ClustalW multiple alignment function with a bootstrap value set to 1000. The phylogenetic tree of the isolated strains was analyzed using 11 standard strains from NCBI (National Center for Biotechnology Information) using the neighbor joining tree method by MEGA 6.0 software program [24,25].

### 2.5. C. perfringens Toxin Detection

All strains identified primarily as *C. perfringens* were examined for *alpha* toxin (*cpa*) genes and enterotoxin (*cpe*) genes using commercially available real-time PCR kits (Kogenbiotech, Seoul, Korea), according to the manufacturers’ instructions. The remaining *beta* toxin (*cpb*), *epsilon* toxin (*etx*), *iota* toxin (*itx*), *beta*2 toxin (*cpb2*) and *NetF* genes were identified by the conventional PCR method. Detection of toxin genes was carried out using primers, probes, and conditions described in a previous study [26].

### 2.6. Antibiogram Test

The antimicrobial susceptibility testing was performed using the MIC Evaluator strips test (E-test) with eight antibiotics (penicillin, amoxicillin/clavulanic acid, ampicillin, tetracycline, vancomycin, meropenem, clindamycin, and metronidazole) according to previous study methods [27,28,29].

## 3. Results

### 3.1. C. perfringens Detection

**Figure 1:**
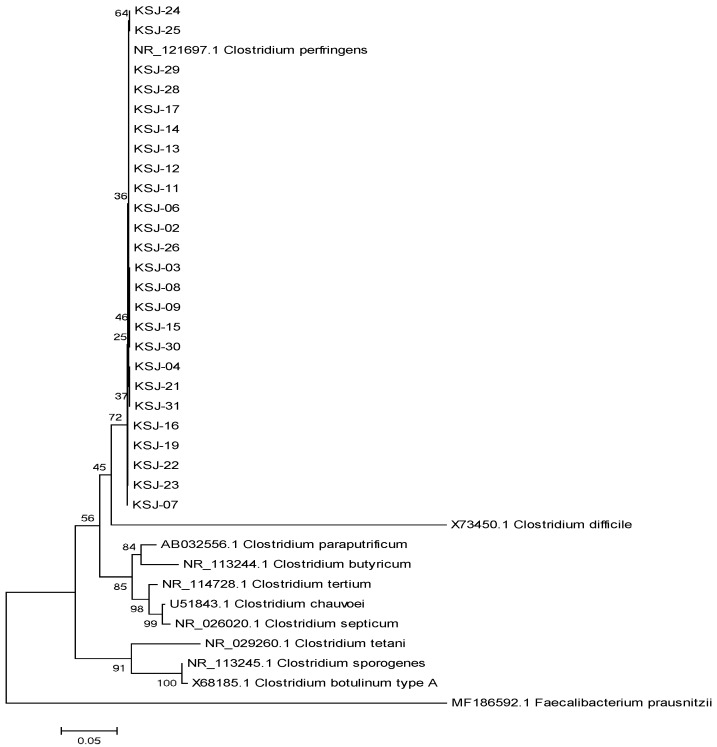
Phylogenetic tree of *C. perfringens* isolated from horse feces and contaminated soil. The nucleotide sequences of all 25 new strains (named KSJ strains) are identical to those of the NR_121697.1 *C. perfringens* (ATCC 13124 *C. perfringens* standard strain). This tree was constructed by the Neighbor-Joining method, MEGA 6.0 program.

Twenty-five (11.1%) *C. perfringens* was isolated from 225 samples tested and 16 (35.56%) of 45 farms. These 25 strains were 100% identical to the nucleotide sequence of *C. perfringens* (ATCC 13124) registered on the NCBI website by using VITEK analysis and 16S rRNA gene sequencing (Figure 1, Table 1).

### 3.2. Toxins and Types of C. perfringens

**Figure 2:**
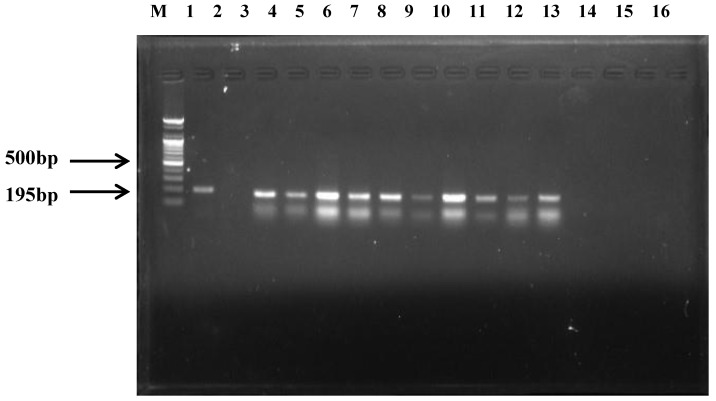
PCR amplification of the *beta*-toxin (*cpb*) from 10 *C. perfringens* isolates. Lane M: molecular size marker (100bp DNA ladder, Bioneer, Korea); Lane 1: standard strain (ATCC 3626) *cpb* gene; Lane 2, 13 to 16; negative control; Lane 3 to 12: *cpb* genes detected from 10 *C. perfringens* isolates.

All the samples were negative for *cpe, etx, itx, NetF* genes and *cpa* gene was detected in 100% (25 of 25) of the samples that were positive for *C. perfringens*. *cpb* and *cpb2* were detected in 40.0% (10 of 25) and 60.0% (15 of 25) of the samples that were positive for *C. perfringens*, respectively (Figure 2, Table 2). Of the 25 *C. perfringens* isolates, 15 (60%) were type A and 10 (40%) were type C. Type C was observed on all seven farms where the foals’ deaths occurred and horse riding tracks at three farms without foals present. None of the isolates were positive for type B, type D, or type E.

### 3.3. Antibiogram Test

The results of the antimicrobial susceptibility testing of *C. perfringens* is shown in Table 3. Briefly, the MIC E-test showed meropenem (96%), ampicillin (92%), amoxicillin/clavulanic acid (84%), and tetracycline (8%) sensitivity.

## 4. Discussion

Due to the extensive size of their colon, horses are more seriously affected by colitis than other animals without an expanded hindgut. Acute enterocolitis in horses is known mainly as *Clostridium difficile, C. perfringens,* larval cyathostomiasis, *Neorickttsia risticii*, and *Salmonella* [30]. Among these infectious agents, *C. difficile* and *Salmonella* are known to be major contributors to acute colitis in horses. Although progress has been made in identifying the causes of acute colitis in horses, the cause is still unknown in approximately 60% of cases [19]. We hypothesized that *C. perfringens* and its production of enterotoxin (*cpe*) and/or beta2 toxin (*cpb2*) are common and important causes of severe colitis in horses [19].

Of 25 *C. perfringens* isolates, 15 (60%) were type A and 10 (40%) were type C. Type C was observed on all the farms where the foals’ deaths occurred. However, it is difficult to conclude that type C is the direct cause of death of the foals. Also, the plasmid DNA extraction has not been carried out perfectly. There are correlation between this experiment and the mortality of the foals. Studies on *C. perfringens* have been carried out in humans and animals, and those of type A, *cpe*, or *cpb2* genes are common. In the case of humans, studies about *Clostridium botulinum* and food poisoning by the *cpe* (enterotoxin) of *C. perfringens* type A, which is a common cause of food poisoning, are progressing. In animals, studies are mainly performed on type A, *cpb2*, *PfoA*, and *NetB* toxins in pigs and chickens [21,22,31,32]. Enterotoxin (*cpe*) was not detected in any of the isolates. The detection of *cpe* gene varies among researchers as well as geographical differences and seasonal variations. This is the first time that the *cpe* gene has been investigated in *C. perfringens* isolated from equine enterocolitis in South Korea. Type C infections have occasionally been recorded as a cause of colitis in adult horses [2]. There was no difference between foals and adults in clinical presentation.

The inclusion of foals is another possible limitation of this study since foals may suffer from viral infections or *C. perfringens* necrotic enteritis toxin B (*NetB*), which could cause a foal’s death, although none were recorded. Autopsies after the deaths of the foals were only conducted on the macro and pathological failures, which also limits the definitive determination of *C. perfringens* as the cause of death of the foals. However, based on this sampled population and acknowledging the limitations of this study, including the absence of a healthy comparison population, we speculate that the cause of death of the foals has considerable relevance to *C. perfringens* type C in South Korea as reported in North America [2,15]. Extensive efforts, including occurrence epidemiology, will be required to identify the unknown causes of severe colitis in horses due to the complexities of case selection, isolating and identifying potential pathogens, and assessing toxin production. In particular, extensive investigations of *cpb2* and *NetB* genes, which are the major causes of enteritis, irrespective of the type of *C. perfringens,* are further required.

Studies are underway to find susceptible antibiotics to treat animals infected with *C. perfringens* [33]. However, antibiotic susceptibility studies on *C. perfringens* in horses in South Korea are limited. Therefore, we performed antibiotic susceptibility tests using 25 *C. perfringens* strains in this study and found that meropenem and ampicillin significantly affected the *C. perfringens* strains. Because *C. perfringens* is a gram-positive bacterium, antibiotics of the carbapenem and penicillin family, which inhibit cell wall synthesis, act strongly. The E-test method has the advantage of obtaining more detailed information because a concentration of 0.002 µg/mL can be used, which is lower than the minimum dilution factor for the general broth dilution method (0.016 µg/mL). This antibiotic test showed no difference in the antibiotic susceptibility of *C. perfringens* according to its type and toxin. Antimicrobial susceptibility testing is very important in the treatment of bacterial diseases. Clearly, vaccination is the most useful method to prevent disease, but if the vaccine is not available, adequate antibiotic treatment in the early stages of the disease may be the next maximized option. Especially in cases of gastrointestinal diseases that affect productivity, rapid treatment must be performed to reduce economic loss. Also, since *C. perfringens* type C infections may cause mortality within one to two days after birth, antibiotics should be considered as a preemptive strategy on farms where foal acute death occurs frequently.

## 5. Conclusions

The purpose of this study was to discover the cause of death of numerous foals in South Korea. *C. perfringens* was isolated from 25 (11.1%) of the 225 horses sampled in this study and from 16 farms (16/45, 35.56%). Of the 25 *C. perfringens* isolates, 15 (60%) were type A and 10 (40%) were type C. Type C was observed on all the farms where the foals’ deaths occurred. These results are the first to identify one of the causes of acute foal death in South Korea and can be used as a criterion to determine the cause of acute foal death and to select effective antibiotics. Extensive efforts, including the occurrence epidemiology, will be required to identify the unknown causes of severe colitis in horses due to the complexities of case selection, isolating and identifying potential pathogens, and assessing toxin production.

## Figures and Tables

**Table 1:** *Clostridium perfringens* identified farms and isolates.

Tract	No. of Farms	No. (%) of *C. perfringens* Identified Farms	No. of *C. perfringens* Isolates	No. of *C. perfringens* Type (A/C)
Jeju-Island *	17	4 (23.53)	9	4/5
Gyeongsang-Province *	5	4 (80.00)	6	5/1
Chungcheong-Province	2	1 (50.00)	1	0/1
Gyunggi-Province	10	4 (40.00)	3	2/1
Jeolla-province	10	2 (20.00)	3	2/1
Gangwon-Province *	1	1 (100.0)	3	2/1
**Total**	**45**	**16 (35.56)**	**25**	**15/10**

* The three tracts where the foal’s death emerged were Jeju Island, Gyeongsang Province, and Gangwon Province.

**Table 2:** Distribution of *cpa, cpb, cpe/etx/itx/NetF, cpb2* genes and types among 25 *Clostridium perfringens* isolated from horses and tracks.

Types	No. (%) of *C. perfringens* Isolates	No. of Isolates from Dead Foals and Tracks	*cpa*	*cpb*	*cpe/etx/itx/NetF*	*cpb2*
A	15 (60.0)	0/0	15/15	0/15	0/15	10/15
C	10 (40.0)	7/3	10/10	10/10	0/10	5/10
Total	25 (100)	7/3	25/25	10/25	0/25	15/25

**Table 3:** Antimicrobial susceptibility testing of 25 *C. perfringens* isolated from horses.

Antibiotics	No. (%) of Isolates		
	Resistance	Intermediate	Susceptible
Penicillin	0(0)	25(100)	0(0)
Amoxicillin/clavulanic acid	3(12)	1(4)	21(84)
Ampicillin	0(0)	2(8)	23(92)
Vancomycin	1(4)	24(96)	0(0)
Tetracycline	0(0)	23(92)	2(8)
Clindamycin	0(0)	25(100)	0(0)
Metronidazole	0(0)	25(100)	0(0)
Meropenem	1(4)	0(0)	24(96)

## References

[1] Deguchi A., Miyamoto K., Kuwahara T., Miki Y., Kaneko I., Li J., McClane B.A., Akimoto S. (2009). Genetic Characterization of Type A Enterotoxigenic *Clostridium perfringens* strains. PLoS ONE.

[2] Diab S.S., Kind H., Moore J., Shahriar M.F., Odani J., Anthenill L., Songer J.G., Uzal F.A. (2012). Pathology of *Clostridium perfringens* Type C Enterotoxemia in Horses. Vet. Pathol..

[3] Li J., Adams V., Bannam T.L., Miyamoto K., Garcia J.P., Uzal F.A., Rood J.I. (2013). Toxin Plasmids of *Clostridium perfringens*. Microbiol. Mol. Biol. Rev..

[4] Nagahama M., Ochi S., Oda M., Miyamoto K., Takehara M., Kobayashi K. (2015). Recent Insights into *Clostridium perfringens* Beta-Toxin. Toxins.

[5] Othani K., Shimizu T. (2016). Regulation of Toxin Production in *Clostridium perfringens*. Toxins.

[6] Uzal F.A., Vidal J.E., McClane B.A., Gurjar A.A. (2014). *Clostridium perfringens* toxins involved in mammalian veterinary diseases. Open Toxinol. J..

[7] Bokori-Brown M., Savva C.G., Fernandes da costa S.P., Naylor C.E., Basak A.K., Titball R.W. (2011). Molecular basis of toxicity of *Clostridium perfringens* epsilon toxin. FEBS. J..

[8] Nakano V., Ignacio A., Lianco L., Bueris V., Sircili M.P., Avila-Campos M.J. (2017). Multiocus sequence typing analyses of *Clostridium perfringens* type A strains harboring tpeLand netB genes. Anaerobe.

[9] Coursodon C.F., Glock R.D., Moore K.L., Cooper K.K., Songer J.G. (2012). TpeL-producing strains of *Clostridium perfringens* type A are highly virulent for broiler chicks. Anaerobe.

[10] Songer J.G. (1996). Clostridial enteric diseases of domestic animals. Clin. Microbiol. Rev..

[11] Gkiourtzidis K., Frey J., Bourtzi-Hatzopoulou E., IIiadis N., Sarris K. (2001). PCR detectionand prevalence of alpha-, beta-, beta2-, epsilon-, iota-, and enterotoxin genes in *Clostridium perfringens* isolated from lambs with Clostridial dysentery. Vet. Microbiol..

[12] Klassen H.L., Molkenboer M.J., Bakker J., Miserez R., Hani H., Frey J., Popoff M.R., Van den bosch J.F. (1999). Detection of the beta2 toxin gene of *Clostridium perfringens* indiarrhoeic piglets in The Netherlands and Switzerland. FEMS. Immunol. Med. Microbiol..

[13] Mahony D.E., Clark G.A., Stringer M.F., MacDonald M.C., Duchesne D.R., Mader J.A. (1986). Rapid extraction of plasmids from *Clostridium perfringens*. Appl. Environ. Microbiol..

[14] Chalmers G., Bruce H.L., Hunter D.B., Parreira V.R., Kulkarni R.R., Jiang Y.F., Prescott J.F., Boerlin P. (2008). Multlocus sequence typing analysis of *Clostridium perfringens* isolates from necrotic enteritis outbreaks in broiler chicken populations. J. Clin. Microbiol..

[15] Howard-Martin M., Morton R., Qualls J.C., MacAllister C.G. (1986). *Clostridium perfringens* type C enterotoxemia in a newborn foal. J. Am. Vet. Med. Assoc..

[16] Bukar A., Mukhtar M.D., Adam S.A. (2008). Current trend in antimicrobial susceptibility pattern of *Clostridium tetani* isolated soil samples Kano. Bayero J. Pure Appl. Sci..

[17] Sanada I., Nishida S. (1965). Isolation of *Clostridium tetani* from soil. J. Bacteriol..

[18] Smith L. (1975). Inhibition of *Clostridium botulinum* by Strains of *Clostridium perfringens* Isolated from Soil. Appl. Microbiol..

[19] Gohari I.M., Arroyo L., MacInnes J.I., Timoney J.F. (2014). Characterization of *Clostridium perfringens* in the feces of adult horses and foals with acute enterocolitis. Can. J. Vet. Res..

[20] Murdoch D.A. (1998). Gram-Positive Anaerobic Cocci. Clin. Microbiol. Rev..

[21] Yoo S.Y., Lee S.U., Park K.Y., Park Y.H. (1997). Molecular Typing and Epidemiological Survey of Prevalence of *Clostridium perfringens* Types by Multiplex PCR. J. Clin. Microbiol..

[22] Kalia V.C., Mukherjee T., Bhushan A., Joshi J., Shankar P., Huma N. (2011). Analysis of the unexplored features of rrs(16S rDNA) of the Genus *Clostridium*. BMC Genom..

[23] Woo P.C., Lau S.K., Chan K.M., Fung A.M., Tang B.S., Yuen K.Y. (2005). *Clostridium* bacteraemia characterized by 16S ribosomal RNA gene sequencing. J. Clin. Pathol..

[24] Fallani M., Rigottier-Gois L., Aguliera M., Bridonneau C., Collignon A., Edwards C.A., Corthier G., Dore J. (2006). *Clostridium difficle* and *Clostridium perfringens* species detected in infant faecal microbiota using 16S rRNA targeted probes. J. Microbiol. Methods..

[25] Hwang J.Y., Cho G.J. (2018). First Identification of *Taylorella equgenitalis* From Genital Tracts of Thoroughbred Horses from the Inland Area of South Korea by Multilocus Sequence Typing. J. Equine Vet. Sci..

[26] Farzan A., Kircanski J., Delay J. (2013). An investigation into the association between cpb2-encoding Clostridum perfringens type A and diarrhea in neonatal piglets. Can. J. Vet. Res..

[27] Andrews J.M., Howe R.A. (2011). BSAC standardized disk susceptibility testing method (version 10). J. Antimicrob. Chemother..

[28] Nachnani S., Scuteri A., Newman M.G., Avanessian A.B., Lomeli S.L. (1992). E-test: A new technique for antimicrobial susceptibility testing for periodontal microorganisms. J. Periodontol..

[29] Thornsberry C. (2016). NCCLS Standards for Antimicrobial Susceptibility Tests. Lab. Med..

[30] Feary D.J., Hassel D.M. (2006). Enteritis and colitis in horses. Vet. Clin. North. Am. Equine Pract..

[31] Lee K.E., Lim S.I., Shin S.H., Kwon Y.K., Kim H.Y., Song J.Y., An D.J. (2014). Distribution of *Clostridium perfringens* isolates from piglets in South Korea. J. Vet. Med. Sci..

[32] Lee Y.J. (2016). Antimicrobial resistance and molecular characterization of *Clostridium perfringens* isolated from chicken. J. Prev. Vet. Med..

[33] Slavic D., Boerlin P., Fabric M., Klotins K., Zothout J.K., Weir P.E., Bateman D. (2011). Antimicrobial susceptibility of *Clostridium perfringens* isolates of bovine, chicken, porcine and turkey origin from Ontario. Can. J. Vet. Res..

